# High Prevalence of Ultrasound Verified Enthesitis in Patients With Inflammatory Bowel Disease With or Without Spondylarthritis

**DOI:** 10.3389/fmed.2021.637459

**Published:** 2021-02-12

**Authors:** Rusmir Husic, Angelika Lackner, Patrizia Katharina Kump, Christoph Högenauer, Winfried Graninger, Christian Dejaco

**Affiliations:** ^1^Department of Rheumatology and Immunology, Medical University Graz, Graz, Austria; ^2^Department of Gastroenterology and Hepatology, Medical University Graz, Graz, Austria; ^3^Department of Rheumatology, Hospital of Brunico (Südtiroler Sanitätsbetrieb-Azienda Sanitaria dell'Alto Adige), Brunico, Italy

**Keywords:** enthesitis, spondyloarthropathies, inflammatory bowel diseases, ultrasound, power Doppler ultrasonography

## Abstract

**Background:** Inflammatory bowel disease (IBD) is closely associated with spondylarthritis (SpA) and enthesitis, as an important feature of SpA, is a common extraintestinal manifestation of IBD. Enthesitis may be clinically silent in a high proportion of patients with IBD without clinical signs or a diagnosis of SpA.

**Objectives:** The aim of this study was to compare the prevalence of ultrasound (US) verified enthesitis in IBD patients with and without SpA, with patients with irritable bowel syndrome (IBS) and healthy subjects (HC) serving as controls.

**Methods:** IBD patients with or without SpA, patients with IBS and HC were prospectively recruited and clinically assessed. Ultrasound examination was performed at 14 entheses. The ultrasound abnormalities were scored according to the Madrid Ankylosing Spondylitis Enthesitis Index (MASEI).

**Results:** We included 33 IBD patients without SpA, 14 IBD patients with SpA, 26 IBS patients and 18 HC. Higher MASEI scores were found in patients with IBD without SpA [median 21.0 range (8.0–53.0)] and IBD associated SpA [33.0 (8–50)] than in IBS patients [10.5 (0–42.0)-*p* < 0.001 for both comparison] and HC [12.0 (2.0–38.0)-*p* < 0.01]. PD, enthesophytes and erosions were more common in patients with IBD with or without SpA as compared to IBS patients and HC. IBD patients with SpA compared to IBD without SpA demonstrated significant higher prevalence of erosion and structural irregularity and consequently significant higher MASEI (*p* < 0.05 for all comparison).

**Conclusions:** Ultrasound verified enthesitis is more common in patients with IBD with or without SpA as compared to patients with IBS or HC.

## Introduction

Enthesitis is defined as inflammation of the insertion of tendons, ligaments, and capsules into bone (1). There are some observations suggesting that repeated mechanical overload or excessive irritation of entheses may lead to enthesal inflammation and given that lower limbs are exposed to higher mechanical load, this might explain why enthesitis primarily involves the lower limbs in patients with spondylarthritis (SpA).

Enthesitis is considered to be the initial lesion in SpA that only secondarily involves bone and synovial tissue leading to spondylitis and arthritis (2). The detection of enthesitis at early stages of SpA might be an interesting window of opportunity to treat patients with this disease.

There is a close link between intestinal disease and SpA. We know that 15% of inflammatory bowel disease (IBD) patients develop peripheral SpA and 36% suffer from sacroilitis (3). Besides, subclinical intestinal inflammation has been identified in up to 60–70% of patients with axial SpA (4, 5). Other patients with IBD might present subclinical or abortive forms of SpA which might only later evolve in clinically manifest SpA.

The prevalence of sub-clinical entheseal involvement in IBD patients, detected with ultrasound, seems to be more common in IBD patients compared to the healthy controls and in sum there is no difference between IBD and SpA (6, 7).

Ultrasound has been used to detect subclinical enthesitis, mainly in lower limbs of patients with SpA (8–10). In patients with psoriasis, another disease that is closely related to SpA and where a proportion of patients develop clinical manifestations of SpA, a high prevalence of subclinical enthesitis was detected by imaging. In psoriasis, it has even been observed that ultrasound verified enthesitis predicted the later development of psoriatic arthritis (11, 12). In patients with IBD, reports about the prevalence and the possible predictive value of subclinical enthesitis are scarce despite the known link between axial, articular, and intestinal inflammation.

The aim of the present study was to investigate the prevalence of enthesitis using sonography in patients with IBD without associated SpA and to compare them to patients with IBD associated SpA, irritable bowel syndrome (IBS) and healthy subjects (HC).

## Methods

We prospectively recruited 47 IBD patients: 33 patients had no clinical symptoms or history of SpA [i.e., no axial, articular, or enthesal pain and 14 patients with confirmed SpA fulfilling the ASAS classification criteria (13)]. All patients fulfilled the diagnostic criteria for IBD (14, 15). As controls, we recruited 26 consecutive IBS patients who all fulfilled the Rome criteria for IBS (16) and 18 HC. All patients and controls gave written informed consent to participate in the study, and the study was approved by the institutional ethics committee (Prot. Number: 23-432 ex 10/11).

### Clinical Assessment

Demographics, level of regular sport activity (defined as sport or physical activity at least once a week) and body mass index (BMI) were determined in all participants. All patients with IBD underwent routine clinical and laboratory examinations and assessment of IBD activity using the Crohn‘s disease activity index (CDAI) for patients with Crohn's disease and the partial Mayo score for ulcerative colitis (17, 18). The Bath Ankylosing Spondylitis Disease Activity Index (BASDAI) was determined in all IBD patients (19). Clinical examination of entheses was conducted in all study participants by a rheumatologist blinded to ultrasound results. Enthesitis was defined as tenderness or swelling at clinical examination at the following sites: entheses of the common extensor tendon at lateral epicondyle, distal insertion of the triceps into the olecranon, quadriceps insertion into the upper pole of the patella, patellar tendon insertion into the lower pole of the patella and into the tibial anterior tuberosity, insertion of the Achilles tendon as well as the insertion of plantar aponeurosis into calcaneal bone.

### Ultrasound Protocol

Ultrasound examination was performed by one of two investigators blinded to clinical results (CD or RH). In total 14 entheses were examined in every subject: bilateral triceps, lateral epicondyles, distal insertion of quadriceps, proximal and distal insertion of patellar tendon, distal insertion of Achilles tendon and plantar fascia using an Esaote MyLab Twice ultrasound device with 18-MHz linear array transducer (20). B-mode and Power Doppler (PD) sonography was performed in a darkened room in which the temperature was held constant at 20°C. PD-settings were standardized accordingly: frequency 9.1 MHz, pulse repetition frequency 750 Hz and medium persistence. The PD-gain was optimized by increasing gain until noise appeared and then reduced just enough to suppress the noise.

**Figure 1:**
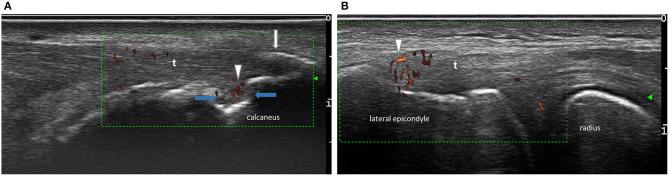
Examples of ultrasound findings. **(A)** Longitudinal scan of a achilles tendon revealing active PD signal (arrows heads), enthesophyt (white arrow), erosion on insertion (blue arrow), **(B)** longitudinal scan of tendon insertion on lateral epicondyl with PD signal (arrow heads), t-tendon.

The following abnormalities were scored according to the Madrid Ankylosing Spondylitis Enthesitis Index (MASEI) as appropriate: Power Doppler (PD) changes, enthesophytes, erosions, enthesal thickening, bursitis, and structural abnormalities (21, 22). According to (MASEI): Structure was considered pathological (score = 1) if there was a loss of fibrillar pattern, hypoechoic aspect, or fusiform thickening of the entheses. Erosions were defined as a cortical breakage with a step-down contour defect at the attachment of entheses at bone and graded with 0 = absent or 3 = present. Fascia and tendon thickness were measured at the point of maximum thickness on the bony insertion and graded with 0 = normal or 1 = thickened according to the reference values published previously. Enthesophytes were defined as calcifications at the entheses insertion into bone and graded with 0 = absent, 1 = small calcification, 2 = clear presence of enthesophyte/calcification, 3 = large calcifications or ossifications (see Figure 1). PD-signals within entheses were scored with 0 = absent or 3 = present. Bursitis was investigated at the level of distal patellar tendon (infrapatellar bursitis) and at the level of Achilles tendon. Bursitis was defined as a well-circumscribed anechoic or hypoechoic area at the site of an anatomical bursa and which was compressible by the transducer. The total (MASEI) score ranges from 0 to 136 for both sides of 12 entheses. In order to calculate the difference of separate ultrasound findings between the groups we calculated a modified MASEI. The MASEI was modified toward the inclusion of the lateral epicondyle given that this site is incorporated in clinical enthesitis scores such as the Leeds Enthesitis Index (LEI) and because previous studies demonstrated a high prevalence of enthesitis at this site in SpA patients (23–26). For this modified score, only the presence =1 or absence = 0 of each abnormality was counted in order to avoid the higher weighting of PD and erosions (presence of any lesions adds three points to the total score) er of enthesophytes (semiquantitative scale of 0–3) as compared to other lesions.

### Statistical Analysis

Statistical analysis was performed using SPSS v22. Descriptive statistics were used to summarize the data. Independent groups of quantitative data were compared with the Student's *T*-Test (parametric) or the Mann-Whitney *U*-test (non-parametric distribution), as appropriate. Correlations analysis was conducted using Pearson *r* (parametric) or Spearman-Rho test (non-parametric data), as appropriate. All statistical analyses were performed using IBM SPSS Statistics (v23.0). Inter-reader agreement between ultrasound examiners was tested in seven patients, in which entheses were scanned by both investigators, and by using the Intra-class correlation coefficient (ICC).

## Results

Patients' characteristics are depicted in Table 1. A greater proportion of the recruited IBD patients (*n* = 47) were male, however we did not observe any gender-based difference of clinical activity of IBD or the MASEI score within the IBD group. The disease duration of IBD showed no significant correlation with IBD activity or with MASEI score. Crohn disease (CD) and Ulcerative colitis (UC) patients yielded the same frequency of therapy with TNFi, Azathioprine or Masalazine. One (7.1%) patient with CD associated SpA and 6 (18.2%) CD patients showed a CDAI score above 150.

**Table 1:** Clinical characteristics of IBD patients and controls.

	**IBD without SpA *n* = 33**	**IBD with SpA *n* = 14**	**IBS *n* = 26**	**HC *n* = 18**
Male, *n* (%)	22 (66.7%)	10 (71.4%)	7 (26.9%) *p* < 0.05^*^	5 (27.8%) *p* < 0.05^*^
IBD type	CD *n* = 27	CD *n* = 8		
	UC *n* = 6	UC *n* = 6		
Age years^‡^	44 (19–62)	45 (21–56)	41 (18–65)	43 (21–58)
Disease duration of IBD in years^‡^	10 (1–30)	7.5 (1–21)		
ESR^‡^	9 (1–45)	10 (3–25)	_	_
CRP^‡^	3.5 (0.6–39.2)	3 (0.1–41.0)	_	_
Anti-TNF therapy, *n* (%)	15 (45)	10 (71.4)	_	_
Azathioprin / Mesalazin, *n* (%)	9 (27) / 7 (21)	1 (7) / 2 (14)		
BASDAI^‡^	2.99 (0.3–7.1)	3.5(1.4–5.9)	_	_
CDAI^†^	87.3 (55.5)	84.9 (50.2)		
Partial Mayo score^†^	4 (1–7)	3.1 (0–5)		
BMI^‡^	24.9 (17.3–34.1)	24.3 (18.8–38.1)	23.6 (16.3–33.2)	22.5 (19.1–31.2)
Sport activity, *n* (%)	13 (39)	6 (43)	19 (73.1)	10 (56)
HLA B 27 positive	-	10 (71%)		

‡ *Median (range)*;

† *mean (standard deviation); BASDAI, Bath Ankylosing Spondylitis Disease Activity Index; BMI, Body Mass Index; CDAI, Crohn's Disease Activity Index; CD, Crohn disease; UC, Ulcerative colitis; CRP, C-reactive protein (normal values 0–5 mg/L); ESR, erythrocyte sedimentation rate (normal values 1–10 mm/1st h); MASEI, Madrid Ankylosing Spondylitis Enthesitis Index; Sport activity defined as sport or physical activity at least once a week; IBD with/without SpA inflammatory bowel disease with or without Spondylarthritis; IBS, irritable bowel syndrome; HC, healthy subjects*.

* *p < 0.05 compared to IBD patients without and with SpA*.

In all examined entheses (*n* = 1,274, all groups), the most common ultrasound abnormalities were enthesophyte/calcification [*n* = 397 (31%)], structural irregularity [*n* = 372 (29%)], increased thickness [*n* = 303 (24%)], bursitis [*n* = 101 (8%)], erosions [*n* = 95 (7%)], and positive PD [*n* = 58 (5%)].

At least one positive PD-signal was observed in nine (64%) patients with IBD and SpA, in 22 (67%) patients with IBD without SpA, in five (19%) patients with IBS and in four (22%) HC. A highest prevalence of PD was observed at lateral epicondyle [39 entheses (67% of all PD positive enthesis)] followed by distal insertion of patellar tendon [*n* = 7 (12%)]. A presence of positive PD at lateral epicondyle did not yield significant difference in group IBD with or without SpA but it was significantly higher compared to IBS and HC (*p* < 0.01). The distribution of PD positive enthesitis among the sites investigated was similar in healthy controls. In patients with IBS, however, lateral epicondyle was more commonly involved than the other enthesis (*p* < 0.05). Comparing prevalence of PD signs at other entheses yielded no difference between groups. There was no difference between involvements of left or right sites.

At least one erosion was observed in 11 (79%, group IBD and SpA), 18 (55 %, group IBD without SpA), 10 (39%, group IBS) patients and 2 (11%) HC. The erosions were found more frequently with comparable distribution in the following enthesis: distal insertion of patellar tendon [22 erosions (23% of all erosions)], triceps tendon [*n* = 19 (20%)] and Achilles tendon [*n* = 18 (19%)]. Bursitis was most frequently observed in distal insertion of patellar tendon 65 (36% out examined enthesis) and in achileon 29 (16% out examined enthesis). Enthesophytes were most commonly observed in the achilleon, followed by lateral epicondyle, triceps tendon, and distal insertion of patellar tendon.

Higher MASEI scores were found in patients with IBD without SpA [median 21.0 range (8.0–53.0)] and IBD associated SpA [33.0 (8–50)] than in IBS patients [10.5 (0–42.0)-*p* < 0.001 for both comparison] and HC [12.0 (2.0–38.0)-*p* < 0.01 for both comparisons]. The MASEI score was significantly higher in IBD with SpA compared to IBD without SpA (*p* < 0.05). Only one patient with IBS did not reveal any US abnormalities according to the MASEI score.

As shown in the Table 2, all separate ultrasound findings were significantly higher in IBD patients with or without SpA than IBS and HC. The modified MASEI score in IBD with SpA [median 29.5 range (9–51)] und IBD without SpA [median 22.0 range (9–39)] did not show significant difference between each other but it was significantly higher compared to IBS [median 10.5 range (2–32)] and HC [median 11.5 (5–33)]. Patients with IBD and SpA demonstrated significantly more erosions (*p* < 0.001) and structural abnormalities (*p* < 0.05) compared to patients with IBD.

**Table 2:** Distribution of ultrasound findings in IBD patients and control population (IBS and HC).

	**Enthesophyte**	**Structure**	**Thickness**	**Bursitis**	**Erosion**	**PD**
IBD with SpA	7 (0–13)	9 (3–14)	6 (0–9)	1.5 (1.6)	3 (0–4)	1 (0–3)
IBD without SpA	6 (2–14)	6 (0–13)^**^	4 (0–8)	2 (0–3)	1 (0–4)^**^	1 (0–2)
IBS	3 (0–12)^*^	0 (0–9)^*^	1 (0–12)^*^	0 (0–3)^*^	0.5 (0–2)^*^	0 (0–2)^*^
HC	4 (0–12)^*^	1.5 (0–7)^*^	1.8 (1.9)^*^	1 (0–4)^*^	0 (0–2)^*^	0 (0–3)^*^

*PD, Power Doppler; Structure, pathological structure of the enthesis; IBD with/without SpA, inflammatory bowel disease with or without spondylarthritis; IBS, irritable bowel syndrome; HC, healthy subjects*.

*Data indicate the median (range) ultrasound score for each abnormalities IBD /IBS per patient or control subjects. Statistical differences were tested with the Wilcoxon test*.

* *p < 0.05 compared to IBD patients without or with SpA*.

** *p < 0.05 compared to IBD patient with SpA*.

No association was found between clinical IBD activity (CDAI and partial Mayo score) and MASEI, nor between clinical IBD activity and erosion-, PD- and enthesophyte subscores. We found no influence on the enthesitis scores by any current therapy or regular sport activities. Out of 25 patients with ongoing TNFi therapy 16 (64%) received Infliximab, eight (32%) Adalimumab and one (4%) Etanercept. We observed no significant difference in the MASEI score between patients receiving the different TNF inhibitors (*p* = 0.75). Besides, we found significant, moderate correlation of BMI with MASEI in the IBS group (*r* = 0.453, *p* < 0.05) and HC (*r* = 0.538, *p* < 0.05).

### Reliability Exercise

The inter-reader agreement for the MASEI was good, revealing an ICC of 0.89 (0.52–0.98).

## Discussion

In the present study we demonstrated that ultrasound verified enthesitis is higher in patients with IBD with or without SpA in comparison to IBS patients and HC. All components of enthesitis were more common in both IBD subgroups.

The most interesting finding of our study is that PD, a sign of active inflammation of entheses, was equally present in patients with IBD with or without associated SpA. Structural changes of entheses including erosions, tended to be higher in IBD with SpA. This also accounted for the higher MASEI score in the latter group. All changes were more common in IBD than in IBS and HC. This indicates that active inflammation of entheses is a common extra-intestinal manifestation in IBD, independent of clinical symptoms of enthesitis (and SpA) as well as IBD activity. Subclinical enthesitis might precede or even predict the development of clinical manifest SpA, as has been the case in psoriasis, but future follow-up studies are required to clarify this issue (11). Structural changes might be the consequence of previous active enthesitis and seem to be linked more closely to clinical features of SpA than PD, but are also independent of current IBD activity.

In our study the prevalence of PD positive enthesis in IBD patients (64%) was higher than reported by Bandinelli at al. who found a positive PD only in 16% IBD patients (6). Possible explanations for the difference are the use of a highly-sensitive 18-MHz linear array transducer in our study compared to a 10-MHz used by Bandinelli. Furthermore, the extensor tendon at lateral epicondyle was frequently a PD positive enthesis in our study and this site was not examined in the Bandinelli study. After the exclusion of lateral epicondyle from the calculation, the distribution of vascularity in IBD patient was in line with other studies like from Rovicso et al. (7). The prevalence of PD and entheseal thickening in IBS and HC in our study was comparable with the data of prevalence of such abnormalities in healthy subjects (27).

The occurrence of a new PsA in a group of patients with asymptomatic enthesitis and in psoriasis was 4.3% during a 2-year observation (12). In CED, 17–39% of patients develop clinically manifest SpA during the course of the disease, however, how many of these cases have asymptomatic enthesitis or other subclinical musculoskeletal manifestation preceding the clinical onset of SpA is unknown and can only be clarified by prospective follow-up studies (28). Our data, however, suggest that the presence of erosion might help in distinguishing between subclinical and definitive SpA in patients with IBD.

Another unresolved issue is the interpretation of clinical and imaging signs of enthesitis in patients with SpA and SpA-related diseases. We know from the present and previous studies that clinical and ultrasound examination of enthesitis only partially overlap. While pain (spontaneous and by palpation) is the most frequent symptom of enthesitis and is also part of the ASAS classification criteria for SpA, it is neither sensitive nor specific for enthesitis, particularly when deep tendon insertions like the plantar facia are affected (29). Nevertheless, little is known about the clinical impact of imaging verified subclinical enthesitis: data is limited on whether patients will develop clinical manifestations of enthesitis later, or if subclinical enthesitis provokes structural damage, or whether it has an impact on the future functionality or quality of life. Further, the response of subclinical, imaging verified enthesitis on anti-inflammatory therapy is unknown. Currently, it is not necessary to treat subclinical enthesitis in IBD patients. Follow-up studies are needed to address these open issues.

While the MASEI included retrocalcaneal and infrapatellar bursitis in the score, bursitis is not considered an elementary lesion of enthesitis according to OMERACT. The OMERACT experts were of the opinion, that bursae are not part of the enthesitis complex and that inflammation affects them only at a later stage of enthesitis when it extends toward the tendon and peri-tendinous structures (30).

While in Rheumatoid arthritis, MSK ultrasound is mainly used to investigate the presence of synovial inflammation, in PsA and SpA it is also applied to investigate enthesitis. Ultrasound verified synovitis in RA predicted the progression of bone erosions and occurrence of a clinical relapse even in the absence of clinical inflammation (31). Whether ultrasound verified synovitis has a similar predictive value in PsA and SpA is unclear. Earlier studies in SpA and PsA demonstrated that ultrasound verified enthesitis does not correlate with clinical signs of enthesitis (20). We previously observed that enthesophytes but neither PD nor clinical tenderness at entheses were linked with radiographic enthesal progression (32). Whether similar result could be found in SpA and whether clinically quiescent but sonographically active enthesitis would predict future clinical worsening are questions that only future research can clarify.

The main strength of our study is the inclusion of IBD patients with and without SpA as well as IBS patients as a relevant control group. While low-grade intestinal inflammation and other factors contribute to the pathophysiology of IBS, there is a clear difference in terms of extra-intestinal enthesal inflammation in comparison to IBD patients, as our data clearly demonstrates. Whether the presence of subclinical enthesitis might also be used for diagnostic purposes (i.e., to differentiate between IBD and IBS patients, needs to be evaluated further). We also took into account the possible influence of external factors such as sport and BMI on the level of enthesitis, however no relevant association was observed in our IBD group.

The main limitations of our study are the limited sample size and the absence of follow-up data which would have been important to analyse the relevance of subclinical enthesitis for future outcomes as stated above. Other limitations are the relatively low level of clinical activity (concerning the intestine) of IBD patients, as well as the heterogeneity of them in regard to disease duration and treatment. All these factors, however, had no influence on the level of ultrasound verified enthesitis in our analyses.

In conclusion, ultrasound verified enthesitis components are more common in patients with IBD with or without SpA as compared to patients with IBS or HC.

## Data Availability Statement

The raw data supporting the conclusions of this article will be made available by the authors, without undue reservation.

## Ethics Statement

The studies involving human participants were reviewed and approved by Ethikkommission der Medizinischen Universität Graz. The patients/participants provided their written informed consent to participate in this study.

## Author Contributions

RH, CD, PK, and CH substantially contributed to study conception and design, acquisition of data, and analysis and interpretation of data. AL was involved in planning, acquisition of data, and supervised the work. RH and CD wrote the paper. All the authors revised the paper and approved the final version of the article to be published.

## Conflict of Interest

The authors declare that the research was conducted in the absence of any commercial or financial relationships that could be construed as a potential conflict of interest.
